# Development and Testing of a Multidimensional iPhone Pain Assessment Application for Adolescents with Cancer

**DOI:** 10.2196/jmir.2350

**Published:** 2013-03-08

**Authors:** Jennifer N Stinson, Lindsay A Jibb, Cynthia Nguyen, Paul C Nathan, Anne Marie Maloney, L Lee Dupuis, J Ted Gerstle, Benjamin Alman, Sevan Hopyan, Caron Strahlendorf, Carol Portwine, Donna L Johnston, Mike Orr

**Affiliations:** ^1^The Hospital for Sick ChildrenToronto, ONCanada; ^2^University of TorontoToronto, ONCanada; ^3^BC Children's HospitalVancouver, BCCanada; ^4^McMaster UniversityHamilton, ONCanada; ^5^Children's Hospital of Eastern OntarioOttawa, ONCanada; ^6^University of OttawaOttawa, ONCanada; ^7^Cundari Group LimitedToronto, ONCanada

**Keywords:** neoplasms, pain, child, adolescent, youth, cellular phone, game

## Abstract

**Background:**

Pain is one of the most common and distressing symptoms reported by adolescents with cancer. Despite advancements in pain assessment and management research, pain due to cancer and/or its treatments continues to be poorly managed. Our research group has developed a native iPhone application (app) called Pain Squad to tackle the problem of poorly managed pain in the adolescent with cancer group. The app functions as an electronic pain diary and is unique in its ability to collect data on pain intensity, duration, location, and the impact pain has on an adolescent’s life (ie, relationships, school work, sleep, mood). It also evaluates medications and other physical and psychological pain management strategies used. Users are prompted twice daily at configurable times to complete 20 questions characterizing their pain and the app transmits results to a database for aggregate reporting through a Web interface. Each diary entry represents a pain case filed by an adolescent with cancer and a reward system (ie, moving up through law-enforcement team ranks, built-in videotaped acknowledgements from fictitious officers) encourages consistent use of the diary.

**Objective:**

Our objective was to design, develop, and test the usability, feasibility, compliance, and satisfaction of a game-based smartphone pain assessment tool for adolescents with cancer.

**Methods:**

We used both low- and high-fidelity qualitative usability testing with qualitative semi-structured, audio-taped interviews and iterative cycles to design and refine the iPhone based Pain Squad app. Qualitative thematic analysis of interviews using constant comparative methodology captured emergent themes related to app usability. Content validity was assessed using question importance-rating surveys completed by participants. Compliance and satisfaction data were collected following a 2-week feasibility trial where users were alarmed to record their pain twice daily on the app.

**Results:**

Thematic analysis of usability interviews showed the app to be appealing overall to adolescents. Analyses of both low- and high-fidelity testing resulted in minor revisions to the app to refine the theme and improve its usability. Adolescents resoundingly endorsed the game-based nature of the app and its virtual reward system. The importance of app pain diary questions was established by content validity analysis. Compliance with the app, assessed during feasibility testing, was high (mean 81%, SD 22%) and adolescents from this phase of the study found the app likeable, easy to use, and not bothersome to complete.

**Conclusions:**

A multifaceted usability approach demonstrated how the Pain Squad app could be made more appealing to children and adolescents with cancer. The game-based nature and built-in reward system of the app was appealing to adolescents and may have resulted in the high compliance rates and satisfaction ratings observed during clinical feasibility testing.

## Introduction

Pain is a common and significant burden to children and adolescents with cancer. This pain may result from cancer, the various cancer treatment methods, or associated procedures, and is known to lower quality of life compared to healthy children [1-6]. Adolescents with cancer (aged 10-18 years) reported that pain was the most distressing symptom of the cancer experience [7,8]. Poorly managed pain has been shown to be additionally problematic as it results in anxiety and distress related to subsequent treatment and negative long-term psychological effects [9,10].

Proper assessment is the essential first step to effectively manage pain experienced by children and adolescents with cancer. Following pain assessment, pain management strategies can be developed, refined, and evaluated to provide the best possible pain relief for adolescents. Still, several barriers prevent appropriate pain assessment for adolescents. There is no well-validated multidimensional tool evaluating the sensory (intensity, quality, and location of pain), affective (emotional effects), and evaluative (pain’s interference with daily activities) dimensions of pain in adolescents with cancer [11]. In addition, existing tools have methodological problems and do not allow for longitudinal assessments in everyday settings [12,13].

Self-report in the form of paper-based approaches has been the most commonly used pain assessment modality [14]. However, patient recall of past experiences involves active reconstruction of events, which can lead to inaccuracies and biases in the reporting of the event [14]. Pain reports can also be influenced by a patient’s affect, as well as the most salient and current state of pain. Finally, patients resort to hoarding (back filling) or completing paper-based pain diaries in longitudinal assessment diaries just prior to returning them to the research center, which reduces the accuracy of collected data [15].

Real-time data capture using electronically-based pain assessments represent a superior method for capturing patient pain data. Wireless mobile devices such as smartphones can: (1) conveniently collect pain reports in natural settings (ie, they are pocket-sized, have large memory capacity, and user-friendly interfaces), (2) be individualized through flexible programming, (3) create time- and date-stamped pain reports to circumvent back-filling, and (4) conveniently upload data to secure electronic databases for review by scientists and clinicians [15]. Recent studies in pediatric [16,17] and adult [18,19] populations have used Internet and mobile device modalities to effectively track and manage health conditions.

Adolescents have adopted mobile technology into their everyday lives in a much quicker and more engaging way than other generations. A recent report indicated that 75% of adolescents aged 12-17 years now own mobile phones [20]. Mobile technology is known to serve five major functions for adolescents - entertainment, information, communication, organization, and support. Of these functions, entertainment and information represent the most frequent uses of technology by adolescents [21]. Regarding the Apple smartphone devices specifically, a 2012 survey of 7700 teenagers found that 40% of teenagers owned iPhones, this was an increase from 23% of teens owning iPhones the year prior [22]. Capitalizing on the popularity of smartphone-based entertainment with adolescents, game-based mobile health applications (apps) may represent an important means to increase compliance with remote monitoring and treatment of health issues.

In the present study, we aimed to design and develop a smartphone-based pain diary app that would be engaging to adolescents with cancer through the gamification of pain assessment recordings. The gamification process involved adolescents playing the role of law-enforcement officers on a special investigative force that hunts down pain. Gamification also included a compliance-based rewards system (ie, proceeding up law-enforcement ranks and receiving videotaped acknowledgements for logging assessments by fictitious officers in the field). We sought to use a user-centered design approach where we actively engaged adolescents in all aspects of the research process from the app’s inception (design and development) through usability and feasibility testing to refine the prototype.

## Methods

### Pain Assessment Questionnaire Development

Pain assessment questions were developed using the e-Ouch electronic juvenile idiopathic arthritis pain diary [23-25] as a template. Expert opinion (ie, captured during a meeting with 10 pediatric oncologists and 10 pediatric pain experts) was used to modify the arthritis diary questions to be cancer pain-specific. The following modifications were made: (1) the body-map was altered to include areas known to be commonly painful in cancer, (2) the addition of a list of possible pain-associated symptoms (eg, nausea), (3) lists of medications and other therapies were changed to include those commonly used to treat cancer pain, and (4) the addition of a list of possible pain sources (eg, treatments, procedures). The final pain assessment questionnaire consisted of 20 questions on the multidimensional (ie, sensory, affective, and evaluative) nature of pain, as well as questions related to pain management strategies used and their effectiveness.

### Pain Squad App Design and Development

#### Pain Squad Design Principles

The design principles included: (1) a multidimensional pain diary to help adolescents track their pain and treatments that help and do not help to reduce pain, (2) a function to alert the research team if pain was moderate to severe for 2 consecutive entries, and (3) the integration of rewards and incentives into the system to sustain engagement with the tool for 2 weeks.

**Figure 1:**
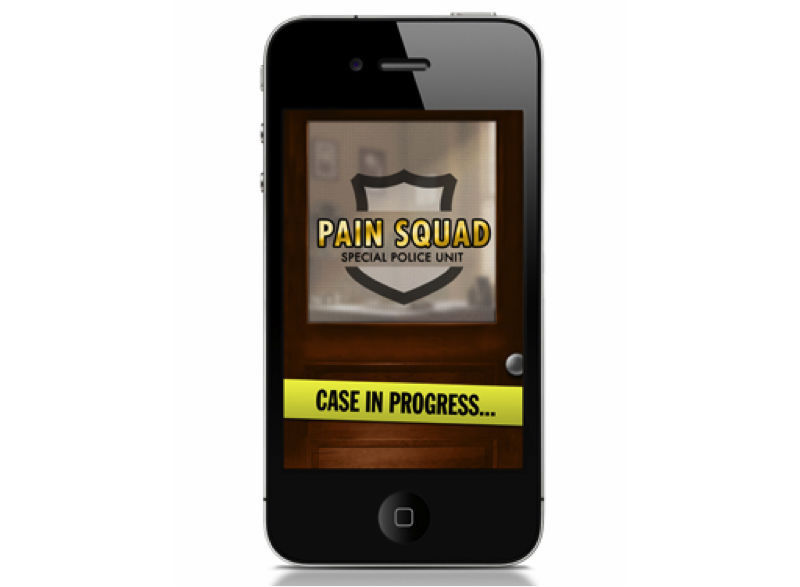
Pain Squad app title screen.

**Figure 2:**
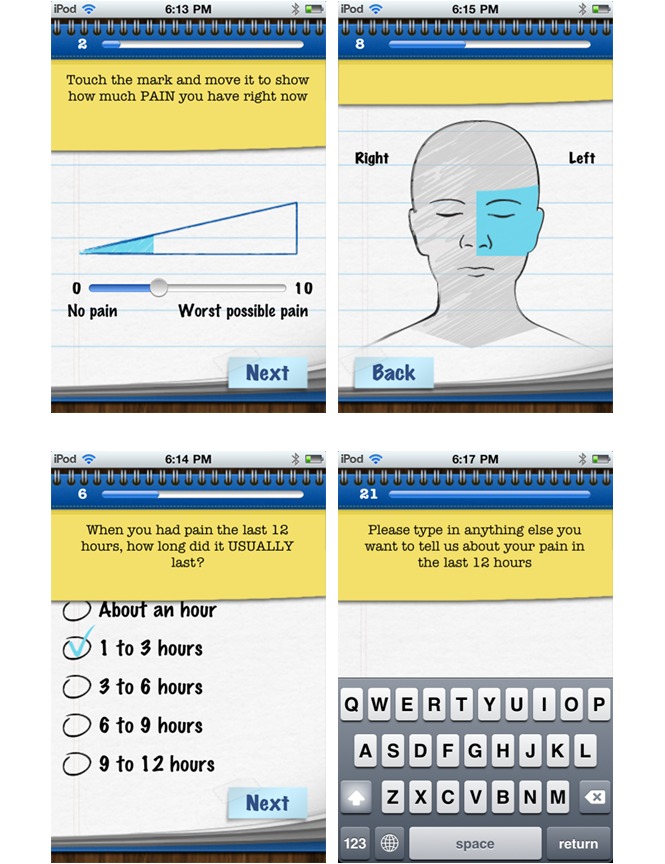
Screenshots of Pain Squad app assessment functionality showing the visual analogue slider scale (top left), the selectable body-map (top right), a multiple-choice question (bottom left), and a free-text question (bottom right).

**Figure 3:**
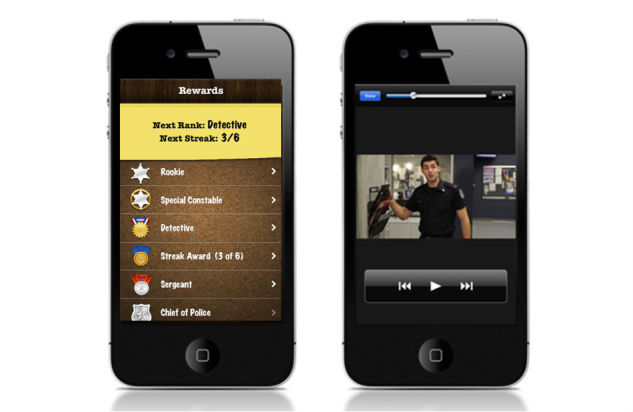
Screenshots of Pain Squad reward system showing compliance-based promotion scheme (left), and videotaped acknowledgement of the adolescent’s compliance with completing pain assessments (right).

Pain Squad, a special police unit, was designed as an incentive for adolescents to use the app (Figure 1). Adolescents were recruited by a special investigative force whose mission was to “hunt down pain and put it behind bars”. Twice a day, adolescents completed a pain-reporting mission by answering questions about pain intensity and location using a simple touchscreen interface. As mentioned, 20 questions about an adolescent’s pain were asked. Questions took the form of: (1) touchable visual analogue slider scales to rate dimensions of pain (pain intensity, pain unpleasantness, pain interference, control over pain) from 0 to 10, (2) a selectable body-map to identify pain locations, (3) multiple choice questions about pain characteristics (duration of pain, causes of pain, pain management strategies used and their effectiveness.), (4) lists of selectable words describing pain and associated symptoms (not shown), and (5) a free-text question to collect additional information adolescents may wish to record (Figure 2). Filling out 3 reports in a row earned a promotion to the next rank in the squad. The promotions were delivered in the form of short videos featuring the stars of Canadian-filmed TV shows Flashpoint and Rookie Blue (Figure 3). The “badge/medal” and video earned by the adolescent through promotion could be re-watched if desired. Pain Squad is not a game in the strictest sense and does not have rules or objectives beyond participation. Instead, the software used features of games such as rewards and achievements. The design concept was refined during low-fidelity usability testing (outlined below).

#### App Development

Once the overarching concept for the app and the app’s design elements were developed in consultation with adolescents (ie, during low-fidelity usability testing), a prototype Pain Squad app was created for high-fidelity usability testing (outlined below). Pain Squad was programmed to be accessible to adolescents using an iPhone 4S. The app was built using client-server architecture. The client component was built in native Apple iOS code and the server component used iBATIS.NET to broker data exchange and an MS SQL Server 2008 database to manage incoming pain assessment data. The system also contained a password-protected Web-interface allowing researchers to create studies, add new study participants, and access results.

Pain Squad used iPhone audible alerts to notify adolescents to complete pain assessments. The system generated 3 alerts for each pain assessment to be completed. The timing of these alerts was tailored to the needs of adolescents by allowing adolescents to choose alarm times from a number of preset options. Data entered by adolescents were stored on the iPhone in an SQLLite database and communicated to the server whenever the application was online. Pain assessment data were not accessible via the iPhone and were transmitted to the server using an encrypted (HTTPS) protocol over a Secure Socket Layer (SSL) connection. The server was hosted at the tertiary care center behind a firewall in a network secure environment. A user name and password were required to access data.

### Participant Characteristics

Adolescents involved in the usability and feasibility testing were recruited from one large university-affiliated tertiary hematology/oncology care center in Toronto, Canada over a 1-year period in 2011-2012. Children and adolescents were eligible to participate if they: (1) were able to speak and read English, (2) were 9 to 18 years old, (3) were diagnosed with cancer, (4) were being seen on an in- or out-patient basis by the oncology team, and (5) had self-reported pain at least once in the last week. Adolescents were excluded if they had severe cognitive impairments or major co-morbid medical or psychiatric illnesses that would preclude pain assessment by self-report. Consent was obtained prior to involvement in any phase of the study and all adolescents completed a brief questionnaire on their demographic characteristics. The research assistants gathered additional demographic and disease-related data from medical charts.

### Phase 1: Usability Testing

#### Phase 1a : Low-Fidelity User-Centered Design

Following the development of the cancer pain assessment questionnaire, app interface designs were trialed with adolescents. A qualitative usability testing approach was used with 3 iterative cycles of semi-structured audiotaped interviews. Fifteen adolescents (5 in each of 3 cycles) were shown paper screenshots of the pain assessment application and were asked what they liked and disliked about the design, premise of the game, and question format. The list of questions was modified during the course of the interview process in light of emerging themes and field notes related to perceived ease of use. Technical problems with the app were recorded by the research assistant. Adolescents were further asked to provide suggestions for improvement. Design elements were modified and new paper screenshots were generated and tested until no further changes were suggested.

#### Phase 1b: High-Fidelity User-Centered Design

Following the development of a fully functional iPhone-based Pain Squad prototype, usability testing (2 iterative cycles) with semi-structured audiotaped interviews was again conducted with 18 adolescents with cancer (21 adolescents were approached to participate and 3 declined). In this phase, a research assistant first provided adolescents with a brief (approximately 5 minutes) demonstration of the Pain Squad app on the iPhone using a standardized pain vignette. Adolescents were then asked to complete the app, recording their own pain while thinking aloud about likes, dislikes, and difficulties with the app. At the end of each session, a research assistant asked a series of standardized open-ended questions related to ease of use, and what adolescents liked or disliked about the app. The research assistant recorded the answers to questions that explored emerging themes and field notes on ease of app use. After the first iterative cycle, changes were made based on themes identified from adolescent opinion. A second iterative cycle was conducted with 8 adolescents, which generated no further recommendations for changes to the app.

Adolescents participating in the high-fidelity testing also rated the content validity of the pain diary questions. To do so, adolescents used a 4-point Likert scale to rate each question from “not important at all” to “very important”.

#### Data Analysis for Usability Testing

In both low- and high-fidelity testing, demographic data were analyzed using SAS version 9.1.3 [26]. Audiotaped usability interviews were transcribed verbatim. All transcripts from the usability testing phases were verified against the tapes and imported into NVivo 8.0 [27] for coding. Field notes taken during the interviews were also transcribed and included in the analytic process. Using grounded theory latent coding [28], data were coded according to the study objective and categorized to reflect emerging themes. Changes to the prototype were made based on feedback from each iterative cycle of testing. In addition, thematic analysis of high-fidelity usability interviews was performed according to adolescent age group (9-12 years and 13-18 years) to identify any age-specific differences in opinion. This analysis was performed in the same manner described above.

### Phase 2: Clinical Feasibility Testing

#### Overview

Following usability testing, a clinical feasibility test was conducted with adolescents with cancer to determine compliance and satisfaction with the app. For this phase, adolescents were trained on app use with standardized pain vignettes. Fourteen adolescents were then loaned an iPhone 4S and asked to complete Pain Squad pain assessments twice daily (in the morning and the evening) for 14 days. Telephone assistance was available to adolescents in case of technical problems. The research team reviewed a summary of each adolescent’s pain reports daily so that patient safety issues could be identified and resolved.

On day 15, adolescents were prompted by the phone using an audible alert to complete the Pain Squad Evaluation Questionnaire on the iPhone, which ascertained likes and dislikes with the Pain Squad app. The Pain Squad Evaluation Questionnaire involved multiple 4-point Likert scales. As an example, selectable options for the question, “How much did you like using the pain diary?” were: “very much liked it”, “liked it okay”, “did not like it or disliked it”, and “did not like it at all”. Data generated from these surveys were used to establish satisfaction and ease-of-use of Pain Squad, the degree to which the app interfered with daily activities as well as information on how long adolescents would be willing to use the diary. The Evaluation Questionnaire also included a free-text question where adolescents were encouraged to enter any other information about the diary they felt important to discuss.

#### Clinical Feasibility Testing Data Analysis

Compliance was defined as 100% when 28/28 entries were completed within the 2-week period. Compliance and satisfaction were analyzed using SAS version 9.1.3 [26]. The level of significance was set at *P*<.05. Comparisons between compliance data generated during feasibility testing were made using *t* tests.

## Results

### Phase 1: Usability Testing

#### Participant Characteristics

Demographic and disease characteristics of adolescents included in the low- and high-fidelity user-centered testing are shown in Table 1. The age of participants in each phase of testing was similar (approximately 13 years). Both male and female participants were included in all phases of the study and participants also had a variety of cancer diagnoses. Time since diagnosis was on average less than 2 years for participants in each study phase.

**Table 1:** Demographics and disease characteristics of adolescents included in low- and high-fidelity usability testing, content validity testing, and feasibility testing.

		Phase 1a: Low-fidelity usability testing (n=15)		Phase 1b: High-fidelity usability and content validity testing (n=18)		Phase 2: Feasibility (n=14)	
Characteristics		Mean (SD)	n (%)	Mean (SD)	n (%)	Mean (SD)	n (%)
**Age (years)** ^**a**^							
		13.9 (1.9)		13.4 (2.9)		13.2 (2.3)	
**Gender**							
	Female		7 (47)		9 (50)		9 (64)
	Male		8 (53)		9 (50)		5 (36)
**Primary diagnosis**							
	ALL		4 (27)		6 (33)		7 (50)
	AML		3 (20)		0 (0)		2 (14)
	Ewing’s Sarcoma		1 (7)		3 (17)		1 (7)
	Non-Hodgkin’s Lymphoma		2 (13)		1 (6)		2 (14)
	Osteosarcoma		2 (13)		4 (22)		1 (7)
	Rhabdomyosarcoma		1 (7)		2 (11)		0 (0)
	Other		2 (13)		2 (11)		1 (7)
**Patient type** ^a^							
	Inpatient		8 (53)		15 (83)		6 (43)
	Outpatient		7 (47)		3 (17)		8 (57)
**Duration of illness (years)** ^a^							
		0.8 (0.8)		1.4 (4.2)		0.8 (0.7)	

^a^at the time of study recruitment

#### Phase 1a: Low-Fidelity User-Centered Design Phase

##### Overview

Transcript analysis from low-fidelity testing revealed 4 distinct themes, which guided the development of the game-based app: (1) a need to change the game theme, (2) the importance of the reward-system, (3) an appreciation for the aesthetics of the app, and (4) changes required to improve ease of use.

##### Change to the Game Theme

During low-fidelity usability testing, and before a capital investment in programming was made, we endeavored to find a theme for the app that was well-liked by adolescents. Adolescents with cancer had difficulty relating to the original game theme and expressed the need to change the game to improve app desirability. The original theme of the game centered around a private detective agency. According to the game, this agency was known as “Gum Shoe” and investigated pain cases. The gumshoe concept fared poorly in usability trials and was considered difficult to relate to by adolescents. In fact, the lack of understanding around the term gumshoe was the most strongly endorsed message in the first iteration of low-fidelity testing. Indeed, none of the adolescents interviewed knew what gumshoe meant. This was articulated by an adolescent who discussed unfamiliarity with the concept.

I don’t know what gumshoe is. I would guess that gumshoe means…just gum on a shoe?

Female, 13 years

**Figure 4:**
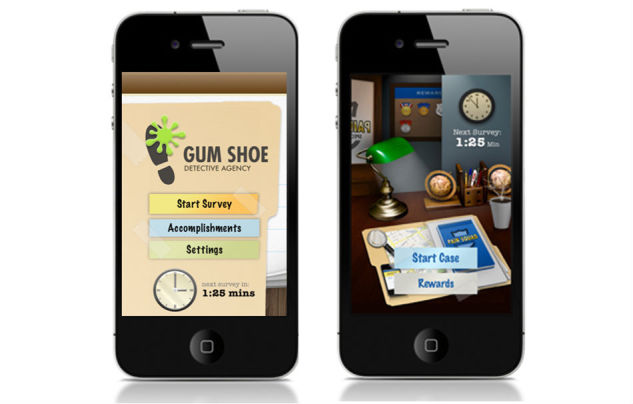
App prototype home screens: the Gum Shoe home screen depicting a detective case file (left), and the Pain Squad home screen depicting the desk of a law enforcement officer (right). Tapping "Start Survey" or "Start Case" begins the pain assessment. Tapping "Accomplishments" or "Rewards" allows review of rank and access to videotaped acknowledgements.

To address this issue, the game theme was changed to “Pain Squad”. Pain Squad took a more modern approach by using a “pain detective” concept and saw adolescents playing the role of law-enforcement officers (Figure 4). This new concept was trialed with adolescents in the second iteration of low-fidelity testing and received overwhelmingly positive endorsements. No further changes to the design theme were required.

##### Importance of the Reward System

Embedded in the Pain Squad app design was a reward system to encourage pain assessment competition. This reward system was highly regarded by adolescents as illustrated below.

I think that [the reward system] is a really good idea! That makes it way more enjoyable. I really like the moving up…idea and the rewards.

Female, 16 years

##### Appreciation for the Game Esthetics

The color scheme, fonts, and graphics used in the Pain Squad app were considered attractive to adolescents, helping to create interest in the app. During low-fidelity testing, adolescents made several positive comments regarding the appearance of the app screenshots:

Green, blue, and yellow are really good colors for this app, for a detective. I like the layout, it’s cool. And I like the font.

Female, 13 years

I would just scroll through and tap on the screen. I like how there are the different choices and the colors. That’s really cool!

Female, 12 years

##### Changes Required to Improve Ease of Use

Based on the screenshots shown, adolescents suggested several changes to improve usability of the game-based app. These changes included adding verbal and numerical anchors to pain assessment scales to guide pain ratings (eg, “no pain” and “worst possible pain”, 0 and 10 respectively) and providing clarification on body parts in the pain location body map. The quote below demonstrates one adolescent’s recommendation that the torso and the abdomen should be combined.

These two parts aren’t clear. I don’t even know what part of the body that is! It’s better to have them together. It’s easier to understand.

Female, 16 years

**Figure 5:**
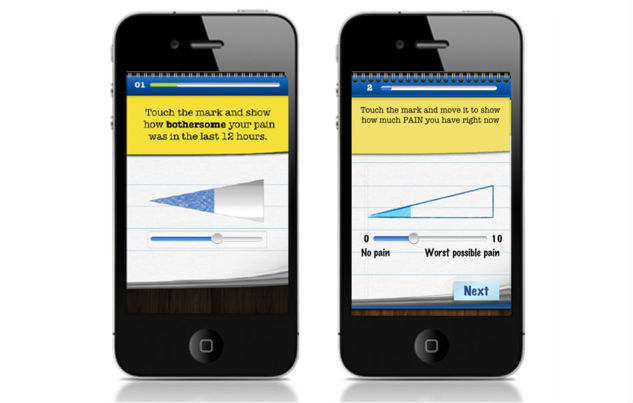
Screenshots of Pain Squad app visual analog scales showing before (left) and after (right) verbal and numerical rating anchors were added.

**Figure 6:**
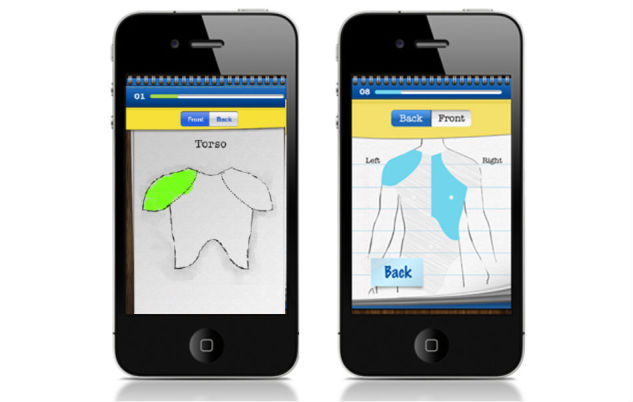
Screenshots of Pain Squad app "body map" showing before (left) and after (right) body parts were combined as recommended by adolescents.

These recommendations were addressed before proceeding to the high-fidelity testing as seen in Figures 5 and 6.

#### Phase 1b: High-Fidelity User-Centered Design Phase and Content Validity Testing

##### Overview

Four themes highlighting the usability of the game-based app were identified through analysis of high-fidelity usability interviews. These themes were: (1) general endorsement of the app, (2) appeal of the game and rewards aspects of the app, (3) ease-of-use of app, and (4) recommendations for improving the user-interface of the app. We found no differences in endorsed themes between younger (9-12 year olds) and older (13-18 year olds) adolescents. These themes therefore represent the convergent perspectives of study participants.

##### General Endorsement of the App

Adolescents overwhelmingly endorsed the app as appealing and fun to use. Every adolescent interviewed said they would use the app daily for an extended period (ie, 2 weeks) if given the option. This satisfaction with the app is illustrated in the following quote from an adolescent with cancer:

Oh cool! It’s so interesting…I liked that you could put down how you felt. Instead of talking to somebody you could just put down how you feel…It was really simple. I liked everything. I think that one, I’d add to my phone!

Female, 11 years

##### Appeal of the Game and Rewards Aspects of the App

The game-like features of the app and the rewards program were also seen as appealing to adolescents with cancer. Adolescents appreciated the ability to role-play as a detective and advance through the law enforcement team ranks.

Oh that’s cool! I liked the way your like a spy trying to solve a case because it made it more interesting.

Male, 10 years

I like that you can see what rank you are and what you need to do [to advance].

Female, 11 years

##### Ease-of-Use of App

All adolescents interviewed also endorsed the ease-of-use of the app. All adolescents found the app to be “easy to understand” and “easy to navigate”. Adolescents also discussed their own familiarly with using electronic devices such as smartphones and did not foresee themselves having any problems using Pain Squad.

##### Recommendations for Improving User Interface of the App

As in the low-fidelity testing, specific suggestions for improvements to the app were made by adolescents. Adolescents in the first iteration of high-fidelity usability testing found that the wording of some of the questions was confusing. For instance, one adolescent did not understand the question, “How much control do you feel you have over your pain?”

It sort of doesn’t really make sense. Like how much control you had? How could you like really have like the control? So I didn’t really like that one.

Male, 9 years

In the first iteration, 4 out of 10 adolescents also discussed the need to be able to select more specific areas on the body location map.

Umm the questions were pretty accurate, though I didn’t like the body map because it wouldn’t let you select exact body parts [in pain].

Male, 11 years

The final first-iteration recommendation by adolescents involved the placement of selectable buttons on the screen. Adolescents found it too easy to advance through questions by accidently tapping on the “Next” button before having time to decide on their answer.

**Figure 7:**
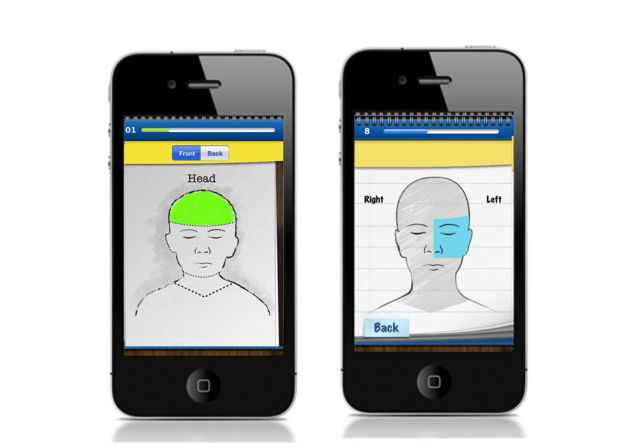
Screenshots of Pain Squad app "body map" showing before (left) and after (right) specificity of selectable pain location was improved.

These issues were addressed by further sub-dividing the body map such that more specific body areas could be selected (Figure 7), simplifying question wording and moving the “Next” button to prevent accidental tapping. No further recommendations for changes were made in the second iteration of testing.

##### Content Validity Testing

Content validity testing affirmed the importance of pain assessment questions for adolescents with cancer. Results indicated that 88% of questions were rated as “important” or “very important” by the majority (>50%) of adolescents. Questions that did not achieve this rating related to the impact of pain on: (1) things adolescents did, (2) schoolwork, and (3) relationships with friends and family.

### Phase 2: Clinical Feasibility Testing

#### Participant and Pain Characteristics

Demographic and disease characteristics are shown in Table 1. Data collected from the feasibility testing provided data on the pain experience of adolescents with cancer. These data showed that average pain reported by an adolescent with cancer at the time of completing the diary was 2.3/10 (SD 1.8). Pain was present for 14% (2/14) of adolescents on every pain assessment completed. Additionally, average worst pain in the 12 hours preceding a reported pain episode was 5.8/10 (SD 1.3). Pain reports received were reviewed by the research team. The team did not need to intervene with any adolescents participants for safety reasons.

#### Compliance

Rates of app compliance were high, with a mean of 81% (SD 22%) of pain assessments completed by adolescents. The relatively large variability (22%) in assessment completion was due in large part to low rates of completion by 2 adolescents. One adolescent completed only 14% (4/28) of the Pain Squad entries and cited that she forgot to keep the iPhone with her as the reason for non-compliance. Another adolescent was hospitalized for an emergency medical complication during the feasibility trial and did not bring the iPhone with her to the hospital. Upon hospital discharge she resumed Pain Squad assessments, completing a total of 57% (16/28) of entries. Average compliance with these 2 adolescents excluded from the analyses was 88% (SD 8%).

There were no differences in mean compliance with assessments between morning and evening reports (82% SD 23% vs 80% SD 23%, *P*=.77) or between week days and weekends (80% SD 24% vs 82% SD 23%, *P*=.84). In addition, compliance with Pain Squad remained high for the duration of the feasibility trial as no difference in assessment completion rates was seen between week 1 and week 2 (84% SD 20% vs 78% SD 29%; *P*=.55). Sub-group analyses of the feasibility trial cohort showed no difference in compliance rates according to gender (females: 79% SD 28%, males: 84% SD 8%, *P*=.68) or initial treatment location (in-patient: 84.5% SD 15.6%, out-patient: 78% SD 27%, *P*=.59).

#### Satisfaction

Data from the post-study Pain Squad Evaluation Questionnaire showed that adolescents enjoyed using the game-based app, found it easy to use and found that it did not interfere with their daily activities. When asked about how much they liked using Pain Squad, 86% of adolescents (12/14) indicated that they “very much liked it” or “liked it okay” and only 14% (2/14) indicated that they “didn’t like it” or “didn’t like it at all”. Regarding the overall appearance of the app, 64% of adolescents (9/14) “very much liked it” and 36% (5/14) “liked it okay”. No adolescents surveyed indicated that they did not like the app design. Regarding ease-of-use, 79% of adolescents (11/14) found the app “very easy” or “easy” to fill out twice a day and 14% (2/14) found it “a little hard” to do. The majority of adolescents also indicated that completing Pain Squad did not affect activities and friendships, with only one participant (7% of respondents) stating that the app “interfered a lot” with these activities. The majority of adolescents (79%; 11/14) reported that 2 weeks was an appropriate amount of time to use the app, but longer periods of usage (ie, greater than 6 weeks) were endorsed by the remainder of respondents.

## Discussion

We present the design and development of a game-based mHealth app to routinely assess pain in adolescents with cancer. We further present data on compliance with completing assessments collected during a 2-week clinical feasibility study. Low-fidelity, user-centered design led to the identification of needed changes to improve the game-based premise of the app and its ease of use (ie, changes to body map, inclusion of anchors to assessment scales). The high-fidelity, user-centered design approach led to the identification of additional needed revisions to the app, but also led to an appreciation of adolescents’ positive reactions to the novel approach of gamification used in this app (video clips from officers in field and graduated reward system). This phase further affirmed the content validity of the pain assessment questions. The feasibility trial demonstrated high rates of compliance and overall satisfaction with the app.

To our knowledge, the present study was the first to report on a user-centered approach to the design and development of a smartphone-based multidimensional pain assessment app for adolescents with cancer. A report on the user-centered design of an mHealth cancer symptom assessment diary for adolescents has been previously published [29]. This study however, did not include adolescents in the design of the interface and did not take a multidimensional approach to the assessment of pain. Our study has used multiple iterative usability cycles (both low- and high-fidelity) to refine the Pain Squad app theme and design. Elucidating the opinions, insights, and recommendations of adolescents from the early stages in app design is critical to ensure a pain assessment tool that is well utilized and clinically helpful. We have also established the content validity of the pain assessment questionnaire through question-importance ratings by end users.

This study was also the first to report on the development and preliminary testing of a game-based mHealth pain assessment tool. We have gamified the concept of pain assessment by: (1) having adolescents with cancer role-playing as officers on the Pain Squad, and (2) rewarding compliance with assessment completion with videotaped acknowledgements and the ability to advance through the law-enforcement ranks. Given the popularity of smartphones with adolescents and the entertainment value of these devices, we reasoned that gamifying health information collection (ie, pain assessment data) using iPhones might improve compliance with reporting. The gamification of the app was highly endorsed by adolescents in both the low- and high-fidelity user-centered design phases. Adolescents helped to refine the game premise, positively rated app aesthetics (eg, colour scheme, font, graphics) and resoundingly endorsed the rewards component of the game.

The clinical feasibility examination showed high compliance rates with the diary irrespective of time of day (ie, morning vs evening) or week (weekday vs weekend). Compliance was also sustained for the duration of the trial, as there were no observed compliance differences between week 1 and week 2. Sub-group analyses also showed no difference in compliance rates between male and female participants or in- and out-patient participants. A systematic review of 62 publications on electronic pain assessment diaries in both children and adults showed compliance rates to be generally high [30]. The authors of the review used backward regression analysis to determine predictors of high compliance and found compliance to be positively associated with shorter diaries, older age of participants, having access to a user’s manual, alarmed reminders, and financial compensation. Additionally important to consider is the clinical context of our study. All of the participants in the feasibility trial were actively being treated for cancer. Cancer treatment is known to be physically exhausting for adolescents [31,32] and could theoretically have adversely effected pain diary completion. Despite this potential adverse impact on reporting, the adolescents completed a large majority of pain assessments on a regular basis.

We propose several possible reasons for the high rates of compliance observed in the present study. First, audible tailored alarms were used to signal participants to complete pain assessments. Audible alarms have been used in previous mHealth assessment studies with adolescents and these studies have also reported high compliance rates [25,33-36]. These alarms act as a direct reminder to participants to log health information. Secondly, we propose that the gamification of the pain assessment app motivated adolescents to complete assessments. Gamification involves the use of videogame components in non-gaming systems to improve user satisfaction [37]. Several previous studies have used reward programs to encourage compliance with mHealth interventions by adolescents [33-35]. However, the reward programming in these studies involved external motivators (ie, money or gift cards given for completing reports). In the present study, we used no such incentive to encourage compliance but observed compliance rates similar to those previous studies. We hypothesize that because of the adolescents’ endorsement of the Pain Squad game premise and virtual rewards system, the app was internally motivating to complete. This is important because the financial feasibility of supplying patients with monetary rewards to log health information, especially over extended periods of time, is limited. Novel mechanisms, such as promotions to the next law-enforcement rank, could inspire improved compliance with health reporting and may ultimately be more practical for use by researchers, clinicians and the health system at large.

As the diary was used twice-daily for 2 weeks, we cannot comment directly on how compliance might be impacted if the app were used for a longer time period. Given that compliance did not significantly change from week 1 to week 2 and previously observed high rates of compliance over weeks to months [30], high compliance rates may be maintained for longer periods. Our data from the Evaluation Questionnaire also indicated that some adolescents were willing to use the app for longer periods (ie, potentially for a period longer than 6 weeks). However, a direct examination of the impact of gamification on long-term compliance with pain diaries remains to be conducted.

Our clinical feasibility test also showed that adolescents with cancer enjoyed using the app over the 2-week period, found it to be attractive and easy to use and felt that it did not interfere with their daily activities. These results are important as they relate to pain management in adolescents with cancer. Because of the complexity of pain management in this group [38-41], accurate and throughout pain assessment is required. Multidimensional pain assessment over extended periods of time (ie, weeks) and multiple times during the day is therefore needed. An electronic pain assessment diary that is well-liked, attractive, and not a burden to complete can assist in collecting important data from adolescents with cancer allowing for improved pain management.

Our study had several limitations that may have tempered interpretation of results and conclusions. First and importantly, we did not directly examine the causes of our observed high compliance rates and could therefore only speculate that they resulted from audible alarms and the gamification of pain assessment. Similarly, should audible alarms and gamification improve compliance, we do not know the degree to which each factor impacted on end user engagement. In addition, our user samples for all phases were drawn from the oncology program at one pediatric tertiary care center, thereby limiting the generalizability of our results. However, with samples as small as 5 end users per iterative usability cycle, the majority of usability problems can be identified [42]. We interviewed each user only once during usability testing and did not verify the results of our thematic analysis with adolescents. Finally, as the Pain Squad app was programmed in Apple iOS code, it was accessible only via Apple devices (ie, iPhone, iPad, iTouch). We are currently exploring options to improve the accessibility of Pain Squad by having it programmed for use on other platforms (eg, Blackberry, Android).

In conclusion, we have demonstrated a user-centered approach to the design and development (including the establishment of content validity) of a novel mHealth pain assessment tool for adolescents with cancer. We also report high rates of compliance and satisfaction with the app as demonstrated in a 2-week clinical feasibility test. We propose that the gamification of the app may have positively impacted compliance and satisfaction and suggest that the use of internal motivators, such as virtual reward systems, is an important area for future mHealth research. Multi-site testing of the validity (including responsiveness) and reliability of the Pain Squad electronic pain diary is currently underway. In addition, future research by our group will focus on using the Pain Squad app as a platform for a clinical decision support system to aid adolescents in making pain management choices. A valid and reliable electronic diary with pain management capabilities has the capacity to result in improved pain management, and ultimately improve quality of life for adolescents with cancer.

## Abbreviations

App: application
SSL: Secure Socket Layer
